# A rare case of Wiskott-Aldrich Syndrome with normal platelet size: a case report

**DOI:** 10.1186/s13256-016-0944-1

**Published:** 2016-06-29

**Authors:** Mohd Farid Baharin, Jasbir Singh Dhaliwal, Smrdhi V. V. Sarachandran, Siti Zaharah Idris, Seoh Leng Yeoh

**Affiliations:** 1 Allergy and Immunology Research Centre, Institute for Medical Research, Jalan Pahang, 50588 Kuala Lumpur, Malaysia; 2 Institute for Medical Research, Jalan Pahang, 50588 Kuala Lumpur, Malaysia; 3 Department of Pediatrics, Hospital Sultanah Bahiyah, KM 6, Jalan Langgar, 05460 Alor Setar, Kedah Malaysia; 4 Department of Pathology, Hospital Sultanah Bahiyah, KM 6, Jalan Langgar, 05460 Alor Setar, Kedah Malaysia; 5 Department of Pediatrics, Hospital Pulau Pinang, Jalan Residensi, 10450 George Town, Pulau Pinang Malaysia

**Keywords:** Wiskott-Aldrich syndrome, Thrombocytopenia, X-linked thrombocytopenia, Recurrent infection, Eczema

## Abstract

**Background:**

Wiskott-Aldrich syndrome is a rare X-linked disorder characterized by microthrombocytopenia, eczema, and recurrent infections. It is caused by mutations of the *WAS* gene. Microthrombocytopenia has been regarded as the key criteria in diagnosing this rare condition. However, in this case report, we describe a case of Wiskott-Aldrich syndrome with normal platelet size.

**Case presentation:**

We report the case of a 9-month-old Malay boy who presented with persistent thrombocytopenia from birth. Serial blood investigations at birth showed he had normal platelet size. His family history revealed two early neonatal deaths in maternal uncles. Spontaneous bleeding was only seen at the age of 3 months. He was initially treated for immune thrombocytopenic purpura and was started on intravenously administered immunoglobulin. His clinical deterioration and poor response to the immunoglobulin raised suspicion for a different underlying pathology. Molecular analysis of the *WAS* gene revealed a missense mutation in exon 10. His parents refused further interventions and defaulted on subsequent follow-up appointments.

**Conclusions:**

A diagnosis of Wiskott-Aldrich syndrome should be considered in any male infant who presents with early onset thrombocytopenia despite an absence of small platelet size, a characteristic feature of Wiskott-Aldrich syndrome.

## Background

Wiskott-Aldrich syndrome (WAS) is a rare X-linked disorder caused by a mutation in the Wiskott-Aldrich syndrome gene (*WAS*) [1]. WAS affects 1–10 babies per million male infants [2]. *WAS* mutations give rise to a wide spectrum of phenotypes with differing severity. The most severe form, referred to as classical WAS, is characterized by a triad of thrombocytopenia, recurrent infections, and eczema, with an increased chance of developing autoimmune conditions and malignancy. The milder form of WAS, known as X-linked thrombocytopenia (XLT), is characterized by thrombocytopenia with absent or mild eczema/immunodeficiency [1, 2]. At present, hematopoietic stem cell transplantation is the only curative therapy for WAS [3]. Nevertheless, gene therapy has recently emerged as a new form of treatment and shows promising results. Further studies on its long-term outcomes and safety are warranted [4].

The *WAS* gene encodes the Wiskott-Aldrich syndrome protein (WASp), which comprises 502 amino acids. WASp is an important regulator of actin cytoskeletal rearrangements. Widely expressed in hematopoietic cells, WASp is involved in various cellular functions including cellular migration and immune synapse formation [5, 6].

Congenital thrombocytopenia and small platelets (mean platelet volume of <5.0 fL in most patients) are the key criteria in diagnosing WAS [7]. In this report, we describe an unusual case of WAS in a Malay boy who presented with early onset thrombocytopenia and a normal platelet size.

## Case presentation

A 9-month-old Malay boy was referred for further investigations in view of persistent thrombocytopenia from birth. He was admitted to the neonatal ward after birth due to borderline prematurity at 35 weeks. A full blood count taken on the first day of life (DOL 0) showed thrombocytopenia with a platelet count of 43,000/μL and a normal mean platelet volume (MPV) of 8.4 fL. Results from a TORCH screen (for *Toxoplasma gondii*, other viruses, rubella, cytomegalovirus, and herpes simplex) were negative. He was treated for presumed sepsis. His platelet count while on our ward ranged from 25,000/μL to 68,000/μL. After discharge, his platelet counts during follow-up appointments at the clinic ranged from 20,000/μL to 30,000/μL and no bleeding tendency was reported.

At the age of 3 months, our patient was admitted due to bloody stools and petechiae (platelet count of 12,000/μL). Following an intravenous infusion of immunoglobulin (IVIG), his platelet count rose and he was discharged. Unfortunately, in his subsequent admissions for epistaxis and bloody stools, his platelet count did not improve even after receiving IVIG. This observation and negative results for an antiplatelet antibody assay raised suspicion for a different underlying pathology and a blood sample was collected from our patient for *WAS* gene analysis. Informed consent for genetic testing was obtained from his parents prior to molecular work. In addition, we also performed a lymphocyte immunophenotyping assay and quantification of his immunoglobulin levels.

**Fig. 1:**
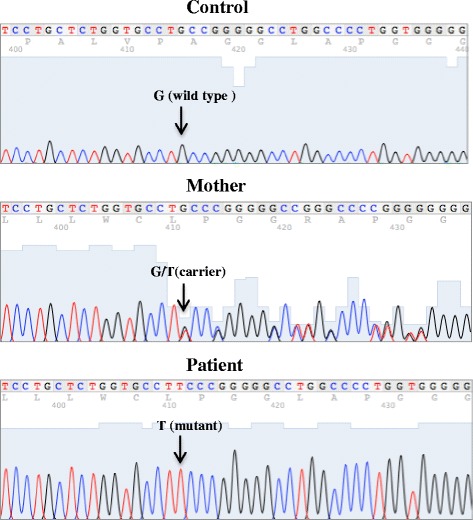
Chromatogram showing DNA sequence in healthy normal control, mother, and patient. There is a G>T mutation in the patient, resulting in a change in the 422nd amino acid from alanine to serine

Our patient was the only male among his siblings. His parents were non-consanguineous. Two of his maternal uncles died during infancy from unknown causes (at ages 1 month and 7 months). None of them was investigated for WAS. A mutation analysis of our patient revealed a c.1264G>T mutation in exon 10 of the *WAS* gene, which led to a change in the 422nd amino acid from alanine to serine. A sequence analysis of his mother showed that she was a carrier. The sequence analysis is shown in Fig. 1.

Lymphocyte immunophenotyping showed elevated total T lymphocytes of 3785 × 10^6^/L (normal range for age 1700–3600 × 10^6^/L), a CD8 count of 1621 × 10^6^/L (normal range for age 800–1200 × 10^6^/L), a low CD4 count of 1356 × 10^6^/L (normal range for age 1700–2800 × 10^6^/L), and normal concentrations of B lymphocyte and natural killer cells. With regards immunoglobulin quantification, he had elevated IgG (1149 mg/dL, normal range for age 400–900 mg/dL), IgA (268 mg/dL, normal range for age 20–60 mg/dL), IgM (126 mg/dL, normal range for age 35–75 mg/dL), and IgE (150 ku/L, normal range for age 2.6 ± 10 ku/L).

Our patient’s disease course deteriorated when he reached the age of 10 months. He started developing severe episodes of hematemesis that required platelet and packed-cell transfusion. Progressive hepatosplenomegaly was also noted, raising suspicion for a malignant transformation of WAS. Bone marrow aspiration was offered, but his parents refused further treatment and then defaulted on their follow-up appointments.

## Discussion

In this report, we describe the case of a patient with WAS who presented with incidental finding of thrombocytopenia at birth. The unusual feature for this report is his normal platelet size despite the thrombocytopenia, a characteristic feature of WAS. There are very few reports describing WAS cases with a normal platelet size. In a previously published case report, Patel *et al*. describe the case of a boy who presented with thrombocytopenia and normal platelet size. Evaluation of WASp expression by flow cytometry showed that he had reduced expression as compared to a healthy control. Molecular analysis of the *WAS* gene revealed a c.862A>T mutation in exon 9 [8]. In a more recent case report, Mantadakis *et al*. described three cases: a pair of twins with a c.854_855insA mutation in exon 9 and a patient with a c.743_743+1delAG mutation in exon 7. In all three cases, WASp expression was undetectable [9]. Our patient demonstrated a novel mutation of c.1264G>T in exon 10. A limitation of our report is the absence of data on WASp expression.

The clinical manifestation of thrombocytopenia in WAS is heterogeneous. In a study involving 154 patients with WAS, the most common bleeding manifestation was either petechiae or purpura, which was seen in 78 % of patients [10]. Bleeding from the gastrointestinal tract, as seen in our patient, only occurred in 28 % of the cases [10]. Splenectomy and IVIG have been shown to improve platelet count in WAS/XLT [11–13]. In our patient, IVIG alone was inadequate to improve his platelet count. Splenectomy could have been beneficial as a supportive treatment in our case, but was not considered because our patient’s platelet counts were persistently very low (<50,000/μL).

The WAS scoring system is used to categorize patients according to disease severity [14]. A score of 1 indicates that the patient has only thrombocytopenia, a score of 2 is given when the patient has thrombocytopenia with additional findings of mild/transient eczema or minor infections. Patients with a score of 1 and 2 are designated as having XLT. Those with treatment-resistant eczema and recurrent infections in spite of optimal treatment receive a score of 3 (mild WAS) or 4 (severe WAS). A score of 5 is given to patients with autoimmune disease or malignancy, regardless of the original score. Using this system, our patient had a score of 1, an indication of the milder XLT variant.

Albert *et al*. investigated the clinical characteristics and long-term outcome of 173 patients with XLT [15]. In their study, there was no association between platelet count and the risk of bleeding. Autoimmunity was seen in 12.1 % of patients and malignancy in 6.9 %. This study suggested that the score of each patient can change over time, and the disease in a patient with XLT may evolve to a more severe classical WAS phenotype. The possibility of malignant transformation in our case could not be confirmed because he was lost to follow-up.

The identification of a mutation in our patient and a carrier state in his mother will enable family counseling in any future pregnancies. An early diagnosis by means of prenatal diagnosis or blood investigations taken in the first few days of life in affected families will not only enable early arrangement of stem cell transplantation, but also avoid potential harmful procedures being administered to infants with WAS, such as the administration of live vaccines and intramuscular injections that may lead to severe infection and bleeding tendencies.

## Conclusions

WAS is a rare disorder with wide clinical manifestations. In the mild WAS phenotype, which is classified as XLT, this condition can be misdiagnosed as immune thrombocytopenic purpura, as seen in our patient. A diagnosis of WAS should be considered in any male child with congenital thrombocytopenia regardless of platelet size, especially when there is a positive family history of early death in male infants.

## Abbreviations

DOL, day of life; IVIG, intravenous immunoglobulin; WAS, Wiskott-Aldrich syndrome; XLT, X-linked thrombocytopenia.
